# Perception of Dental Professionals towards Biostatistics

**DOI:** 10.1155/2014/291807

**Published:** 2014-10-28

**Authors:** Manu Batra, Mudit Gupta, Subha Soumya Dany, Prashant Rajput

**Affiliations:** ^1^Department of Public Health Dentistry, Teerthanker Mahaveer Dental College and Research Centre, Moradabad, India; ^2^Department of Oral Medicine & Radiology, Uttaranchal Dental & Medical Research Institute, Dehradun, Uttarakhand, India; ^3^Department of Public Health Dentistry, Kothiwal Dental College and Research Centre, Moradabad, India

## Abstract

Biostatistics is becoming an integral part of dental sciences. Awareness regarding the subject is not thoroughly assessed in the field of dentistry. So the study was conducted to assess dental professionals' knowledge, attitude, and perception toward biostatistics at an academic dental institution. An anonymous cross-sectional questionnaire survey was conducted among all the faculty and postgraduate students of two dental colleges in Moradabad, Uttar Pradesh. The responses were assessed on 5-point likert scale. The survey response rate was 73.71%. Two-thirds of respondents believed biostatistics to be a difficult subject and at the same time half of them did not consider it to be more difficult than other subjects in dentistry. Females were less competent than males in applying biostatistical skills which was found to be statistically significant. Results suggested that dentists with research or academics as an adjunct to their clinical practice had better command over the subject. The current study shows that there is lack of command over the subject of biostatistics among dental professionals although they were aware of its importance in dentistry. There is a need of changing the training pattern of biostatistics for dental professionals which would make them confident enough to apply biostatistics in their clinical practice.

## 1. Introduction

It has become a tough task to remain updated with the dental literature as the number of dental publications has increased several folds [1]. Nowadays it has become routine work for the clinician and academicians to review articles that might have an impact on patient care, as well as articles describing changes in the philosophy of dental health care delivery and new vistas in dental research and education.

Most of the articles are accompanied by statistics. Presumably, the statistical analysis is presented by the authors to either validate or question their findings or conclusions of particular investigations or the assertions they have made in these articles.

Statistics have become an important tool or instrument in dental research. As such, when they are used judiciously and appropriately, they can shed additional light and clarity upon subjects under study. On the other hand, any tool or instrument can be misused. This can result in confusion and validation because the results or conclusions of the studies are without merit. In these instances, statistics have been used, as we have heard, “as a drunken man uses a lamppost—for support rather than illumination.”

Articles in dental publications frequently include both descriptive and inferential statistics. To a great extent, authors (and editors) of dental publications rely upon professional statisticians who are experts in the field and have more than just a casual familiarity with dentistry. There is a need to figure out the associated dilemmas in concluding the statistical data. The proper use and understanding of statistics are necessary to differentiate between the “gold” and the “dross” [1].

A study of doctors' knowledge of these statistical expressions has previously been published [2]. The authors of this study concluded that the statistical knowledge of most doctors is so limited that they cannot be expected to draw the right conclusions from statistical analysis. Dentists' need for a basic statistical knowledge to a great extent is similar to doctors' [3].

Statistical knowledge can lead to the attitude of dental professionals towards the subject, but as a whole it depends on their perception about the same. So the objective of this survey was to evaluate the knowledge, attitude, and perception of dental professionals towards the biostatistics as it will further help in improving the teaching methods of the subject.

## 2. Subjects and Methods

An anonymous cross-sectional questionnaire based survey was conducted in two dental colleges of Moradabad, India, namely, Kothiwal Dental College and Research Centre and Teerthanker Mahaveer Dental College. All the faculty members and postgraduate students were considered eligible for this survey. For this a list of all the faculty members and postgraduate students was made along with details of their telephone numbers and e-mail addresses. Questionnaire was administered to each of the eligible members in person by the researcher. A week time was given to all the participants to fill up the questionnaire and in between reminders were sent either by telephonic conversation or by e-mail after which they were collected by the researcher only. Questions using 5-point Likert scales which were adapted from validated existing surveys that addressed medical clinicians' attitudes toward biostatistics were used [4–6]. Questions from the validated questionnaire [6] were taken directly with slight modifications being made to match it for the dental professionals. Questionnaire (Table 1) consisted of 18 questions pertaining to their knowledge about biostatistics. Demographic data were collected on sex, department, academic position, and career focus. Data were primarily analysed by PSPP version 0.6.2. Associations between responses to certain questions and demographic factors were analysed with the chi-square test level of statistical significance being set as 0.05. This study was approved by the Ethical Committee of Kothiwal Dental College and Research Centre and consent to conduct the study was obtained from the Heads of both of the above-mentioned institutions.

## 3. Results

The survey response rate was 73.71% (258/350). Response rates differed by academic position, with a significantly lower response rate among teaching faculty (54.73% versus 87.62% among postgraduate students; *P* < 0.001). One hundred and forty-two (78.02%) of 182 eligible male participants and one hundred and sixteen (69.05%) of 168 eligible female participants responded. Out of total respondents faculty members were 81 (31.4%) and 177 (68.6%) were postgraduate students. Most of the study participants (57.7%) focused on academic clinical careers followed by research work (Table 2).

Responses to each of the individual survey questions are presented in Table 3. Biostatistics was believed to be a difficult subject by 64.3% respondents, and also 53.5% subjects disagreed that it is more difficult than other subjects in dentistry. Most of the respondents (63.2%) believed that it would be helpful for them if the teachers/consultant biostatisticians whom they are consulting for statistical help have some knowledge of dentistry so that they could understand their needs. 69.8% respondents agreed that knowing biostatistics will benefit their career.

Only 26.0% of respondents reported that their training in biostatistics was adequate for their needs whereas 27.2% felt that their current level of training in biostatistics in dentistry is adequate and only 23.6% thought their biostatistics coursework had been taught effectively. A total of 30.2% of respondents agreed that they could identify when correct statistical methods had been applied in a study and 31.3% of respondents believed they could design their own research projects with confidence, and 17.5% respondents were confident that they can conduct their own statistical analyses with confidence.

Around 71.2% of respondents agreed that biostatistics should be an integral part of research and the same proportion of respondents feels that it is a necessary skill for research purposes. The majority of respondents (71.3%) felt that knowledge of biostatistics is necessary while evaluating dental literature.

More female respondents (70.7%) in comparison to male respondents (56.3%) felt that biostatistics was a tough subject (*P* = 0.03) and very few female respondents (13.8%) were confident that they could apply statistical analysis of their own; in comparison to this, 20.4% male respondents were confident that they could conduct their own statistical analysis (*P* = 0.01).

There was no statistical difference in their agreement on biostatistics being a difficult subject (*P* = 0.1) when comparison was made between different academic positions whereas there was a highly significant difference among the proportion of staff (39.5%) in comparison to PG students (27.1%) regarding the confidence of designing their own research projects (*P* < 0.001). More of staff respondents (29.6%) were confident that they can apply statistical analysis on their own in comparison to 13.0% PG students (*P* < 0.001).

Career focus affected the response of the questions as there were highly statistically significant differences among the clinical academic respondents, clinical nonacademic respondents, and research respondents. There were very few respondents who had other career focuses which are not considered here. Apart from research respondents the rest of respondents felt that biostatistics was a tough subject (*P* < 0.001). Research respondents (48.3%) and clinical academic respondents (29.1%) were confident of designing their own study in comparison to clinical nonacademic respondents (17.8%) agreeing for the same. Research respondents (41.4%) were much more confident in conducting their own statistical analysis in comparison to other respondents (*P* = 0.05) (Table 4).

## 4. Discussion

The results of our study suggest that though more than half of the respondents understand the importance of the subject and also believe that knowing the subject will be beneficial for their career along this the same amount of respondents feels that biostatistics is a difficult subject when placed next to other disciplines of dentistry; it is relegated to a supporting role. This indicates that teaching of biostatistics as a separate entity is unlikely to change the scenario. So more integrated approaches that demonstrate how biostatistics can affect patient care decisions, as suggested in other studies, may be more effective [7, 8], which will revive the importance of biostatistics in clinical decision making, which requires both knowledge of biological processes and interpretation of research reports [9]. In addition, the faculty who are teaching statistics in the workshops and CDE programs mostly have background of pure statistics and do not have the training of teaching methods of conveying statistical concepts to the dental professionals [10].

The study showed that a very small proportion of respondents were confident enough in designing their own research projects and performing statistical analysis. Our results were in agreement with Wulff et al. [2] who also concluded that statistical knowledge of doctors was unsatisfactory when it came to biostatistics. Altman and Bland [11] also highlighted the same inference. There was statistical difference by gender while considering the application of statistical tests: males were more confident than females. Studies on math performance and sex differences demonstrate that men perform better in mathematics as compared to females [12] which can elucidate the results of our study. Career focus and academic position also affected the responses of the above discussed question. The respondents who had research, or clinical along with academic career, focus were much more confident about the application of statistical tests than respondents who had clinical nonacademic career focus. Former group were more updated as they are actively encountering biostatistics in their research and academic sessions with their students quite frequently. Regarding academic position, it is quite obvious that with greater experience the faculty over scores postgraduate students in having confidence over subject.

Respondents' evaluation of biostatistics as an important element of evidence based dentistry suggested that EBD may represent an ideal vehicle for improved teaching of biostatistics. Unfortunately, many biostatisticians appear relatively unaware of the EBD movement and do not use the successful EBD integration techniques in their teaching. Similarly, EBD has not fully embraced the teaching of statistical methods, preferring to focus instead on epidemiological principles [13, 14].

Possible solutions to the limitations in teaching biostatistics identified by our respondents can be incorporation of problem based learning into biostatistics course which can be quite helpful. There should be a student-centred approach, substantial student involvement/engagement, and heavy emphasis on application [15]. For dental students encountering statistics for the first time, with little prior knowledge, it is important that they encounter it in the context that they will be using it during their subsequent research careers. Need of the hour is to move from didactic learning towards more problem-oriented and practical approach, that is, problem based learning.

Our study results could be affected by response bias as the demographic data was not available for nonrespondents. Another limitation is that the survey has been limited to a single private dental institution so it cannot be generalized as there would be variations in responses of government institutions. This is the first study representing the perceptions of dental professionals towards biostatistics. Further research is needed to identify effective methods that will transform their perceptions towards the subject.

## 5. Conclusion

The current study shows that there is lack of command over the subject of biostatistics among dental professionals although they were aware of its importance in dentistry. The fact that only 23.6% of respondents agreed or strongly agreed that their biostatistics courses were taught effectively suggests that there is a need of changing the training pattern of biostatistics for dental professionals which would make them confident enough to apply biostatistics in their clinical practice.

## Figures and Tables

**Table 1:** Questionnaire to assess perceptions towards biostatistics.

**General information**					
Gender:	Department:				
Academic position:					
Career focus: Clinical, nonacademic □ Clinical, academic □	Research □		Others □		
	Strongly disagree	Disagree	Neutral	Agree	Strongly agree

General perceptions					
Biostatistics is a difficult subject					
Biostatistics is more difficult than any other subject in dental training					
Biostatistics would be more helpful for teachers and consultants if they understood dentistry					
Within the dental field, biostatisticians have high status					
It would benefit my career to better understand biostatistics					

Perceptions of knowledge and training					
My training in biostatistics is adequate for my needs					
The current level of training in biostatistics in dentistry is adequate					
My previous biostatistics coursework was taught effectively					
I am able to tell when the correct statistical method has been applied in my study					
I am able to design my own research projects with confidence					
I am able to conduct my own statistical analyses with confidence					

Perceptions of biostatistics and research					
Biostatistics should be an integral part of most research					
Biostatistics is a necessary skill for a clinician involved in research					
Biostatistics is a necessary skill for a clinician not involved in research					
Biostatisticians are not necessary for most research					

Perceptions of biostatisticians and evidence based dentistry					
Biostatistics is an important part of evidence based dentistry					
Knowledge of biostatistics is necessary when evaluating dental literature					
Evidence based dentistry is important for clinical practice					

**Table 2:** Distribution of survey respondents.

Demographic	Number (%) of respondents
Gender	
Male	142 (55.04%)
Female	116 (44.96%)
Academic position	
Staff	81 (31.4%)
PG students	177 (68.6%)
Career focus	
Clinical, academic	149 (57.7%)
Clinical, nonacademic	45 (17.5%)
Research	58 (22.5%)
Others	6 (2.3%)
Department	
Conservative and endodontics	32 (12.4%)
Oral medicine and radiology	28 (10.8%)
Oral pathology and microbiology	27 (10.5%)
Orthodontics	30 (11.6%)
Oral and maxillofacial surgery	25 (9.78%)
Pedodontics	26 (10.1%)
Periodontics	29 (11.2%)
Public health dentistry	27 (10.5%)
Prosthodontics	34 (13.2%)

^*^Some percentages do not total 100 because of rounding.

**Table 3:** Survey responses of dentists towards biostatistics.

Question			Number (%)		
	Strongly disagree	Disagree	Neutral	Agree	Strongly agree
General perceptions					
Biostatistics is a difficult subject	16 (6.2)	58 (22.5)	18 (7.0)	140 (54.2)	26 (10.1)
Biostatistics is more difficult than any other subject in dental training	34 (13.2)	104 (40.3)	40 (15.5)	53 (20.5)	27 (10.5)
Biostatistics would be more helpful for teachers and consultants if they understood dentistry	17 (6.6)	13 (5.0)	65 (25.2)	119 (46.f1)	44 (17.1)
Within the dental field, biostatisticians have high status	17 (6.6)	31 (12.0)	92 (35.7)	103 (39.9)	15 (5.8)
It would benefit my career to better understand biostatistics	5 (1.9)	16 (6.2)	57 (22.1)	138 (53.5)	42 (16.3)

Perceptions of knowledge and training					
My training in biostatistics is adequate for my needs	13 (5.1)	101 (39.1)	77 (29.8)	62 (24.1)	5 (1.9)
The current level of training in biostatistics in dentistry is adequate	33 (12.8)	102 (39.5)	53 (20.5)	69 (26.8)	1 (0.4)
My previous biostatistics coursework was taught effectively	11 (4.3)	105 (40.7)	81 (31.4)	58 (22.5)	3 (1.1)
I am able to tell when the correct statistical method has been applied in my study	13 (5.1)	94 (36.4)	73 (28.3)	74 (28.7)	4 (1.5)
I am able to design my own research projects with confidence	20 (7.8)	95 (36.8)	62 (24.1)	78 (30.2)	3 (1.1)
I am able to conduct my own statistical analyses with confidence	16 (6.2)	119 (46.1)	78 (30.2)	43 (16.7)	2 (0.8)

Perceptions of biostatistics and research					
Biostatistics should be an integral part of most research	3 (1.1)	16 (6.2)	58 (22.5)	124 (48.1)	57 (22.1)
Biostatistics is a necessary skill for a clinician involved in research	4 (1.6)	17 (6.6)	53 (20.5)	119 (46.1)	65 (25.2)
Biostatistics is a necessary skill for a clinician not involved in research	13 (5.1)	95 (36.8)	104 (40.3)	41 (15.9)	5 (1.9)
Biostatisticians are not necessary for most research	62 (24.1)	104 (40.3)	57 (22.1)	31 (12.0)	4 (1.5)

Perceptions of biostatisticians and evidence based dentistry					
Biostatistics is an important part of evidence based dentistry	13 (5.1)	16 (6.2)	32 (12.4)	140 (54.2)	57 (22.1)
Knowledge of biostatistics is necessary when evaluating dental literature	5 (1.9)	20 (7.8)	40 (15.5)	135 (52.3)	58 (22.5)
Evidence based dentistry is important for clinical practice	11 (4.3)	14 (5.4)	37 (14.3)	141 (54.8)	55 (21.2)

^*^Some percentages do not total 100 because of rounding.

**Table 4:** Analysis of perception of knowledge by gender, career focus, and academic position.

				I am able to		I am able to conduct	
		Biostatistics is a difficult		design my own		my own statistical	
		subject		research projects		analyses with	
				with confidence		confidence	
			*P* value		*P* value		*P* value

Gender	Male	80 (56.3)	0.03	53 (37.3)	0.06	29 (20.4)	0.01
	Female	82 (70.7)		22 (18.9)		16 (13.8)	

Academic position	Staff	46 (56.8)	0.1	32 (39.5)	<0.001	24 (29.6)	<0.001
	PG students	109 (61.6)		48 (27.1)		23 (13.0)	

Career focus	Clinical academic	114 (76.5)	<0.001	44 (29.5)	<0.001	21 (14.1)	0.005
	Clinical nonacademic	40 (88.9)		8 (17.8)		6 (13.3)	
	Research	4 (6.9)		28 (48.3)		24 (41.4)	

^#^ *P* values based on chi-square test.
